# The effects of traffic-related air pollutants on chronic obstructive pulmonary disease in the community-based general population

**DOI:** 10.1186/s12931-021-01812-x

**Published:** 2021-08-03

**Authors:** Hui-Tsung Hsu, Chih-Da Wu, Mu-Chi Chung, Te-Chun Shen, Ting-Ju Lai, Chiu-Ying Chen, Ruey-Yun Wang, Chi-Jung Chung

**Affiliations:** 1 Department of Public Health, College of Public Health, China Medical University, No. 100, Sec. 1, Jingmao Rd., Taichung, 406040 Taiwan; 2 Department of Geomatics, National Cheng Kung University, Tainan, Taiwan; 3 National Institute of Environmental Health Sciences, National Health Research Institutes, Miaoli, Taiwan; 4 Division of Nephrology, Department of Medicine, Taichung Veterans General Hospital, Taichung, Taiwan; 5 Division of Pulmonary and Critical Care Medicine, Department of Internal Medicine, China Medical University Hospital, Taichung, Taiwan; 6 Department of Medical Research, China Medical University Hospital, Taichung, Taiwan

**Keywords:** PM_2.5_, O_3_, Chronic obstructive pulmonary disease, Land-use regression model, Air pollution

## Abstract

**Background:**

Previous studies have shown inconsistent results regarding the impact of traffic pollution on the prevalence of chronic obstructive pulmonary disease (COPD). Therefore, using frequency matching and propensity scores, we explored the association between traffic pollution and COPD in a cohort of 8284 residents in a major agricultural county in Taiwan.

**Methods:**

All subjects completed a structured questionnaire interview and health checkups. Subjects with COPD were identified using Taiwan National Health Insurance Research Databases. A hybrid kriging/LUR model was used to identify levels of traffic-related air pollutants (PM_2.5_ and O_3_). Multiple logistic regression models were used to calculate the prevalence ratios (PRs) of COPD and evaluate the role played by traffic-related indices between air pollutants and COPD. The distributed lag nonlinear model was applied in the analysis; we excluded current or ever smokers to perform the sensitivity analysis.

**Results:**

Increased PRs of COPD per SD increment of PM_2.5_ were 1.10 (95% CI 1.05–1.15) and 1.25 (95% CI 1.13–1.40) in the population with age and sex matching as well as propensity-score matching, respectively. The results of the sensitivity analysis were similar between the single and two pollutant models. PM_2.5_ concentrations were significantly associated with traffic flow including sedans, buses, and trucks (p < 0.01). The higher road area and the higher PM_2.5_ concentrations near the subject’s residence correlated with a greater risk of developing COPD (p for interaction < 0.01).

**Conclusions:**

Our results suggest that long-term exposure to traffic-related air pollution may be positively associated with the prevalence of COPD.

**Graphical abstract:**

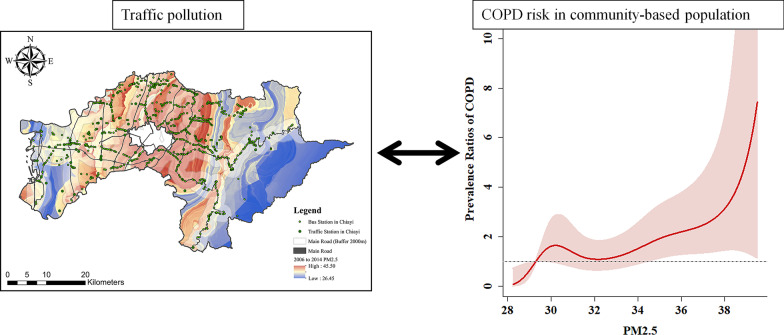

**Supplementary Information:**

The online version contains supplementary material available at 10.1186/s12931-021-01812-x.

## Introduction

Chiayi County is located in the southwest part of Taiwan, and the Tropic of Cancer runs through it. It has the third largest agricultural land area in Taiwan. Therefore, the population density and industrial area of Chiayi County are not very dense. However, according to the environmental resource database of the Taiwan Environmental Protection Administration (TWEPA, 2020), the population of Chiayi County decreased from 563,365 in 2001 to 507,068 in 2018. Nevertheless, the number of vehicles (including cars and motorcycles) increased from 448,824 in 2001 to 523,851 in 2018. In other words, the number of vehicles owned by each person increased from 0.8 in 2001 to 1.03 in 2018; indicating that everyone in Chiayi County has one vehicle. Under such circumstances, the air pollution caused by traffic and its effects on human health is a subject worth exploring. According to data from the Taiwan Ministry of Health and Welfare, the age-standardized mortality rate of chronic lower respiratory tract in Chiayi County in 2009 was 14.5 per 100,000 persons, and by 2018, it rose to 16.3 per 100,000 persons. In contrast, Taiwan’s national data showed that in 2009, it was 14.9 per 100,000 persons, and by 2018, it had dropped to 12.8 per 100,000 persons (Taiwan Ministry of Health and Welfare, 2020). These data show that the mortality of respiratory disease in Chiayi County is gradually worsening compared with that of the whole country.

According to data from the Taiwan Air Quality Monitoring Network operated by Taiwan Environmental Protection Administration (2020), the most important pollutant in Chiayi County in the past six years (2014–2019) was PM_2.5_. During these periods, there were approximately 28% of daily mean PM_2.5_ concentrations in Chiayi County that exceeded Taiwan’s air quality standard (35 μg/m^3^). It is suggested that traffic is one of the most important contributors of PM_2.5_ in Chiayi County.

Particulate matter (PM) is a complicated mixture of solid and liquid particles consisting of consists of organic chemicals, metals, sulfate, nitrate, and ammonium. In addition, ozone (O_3_) is a well-known strong oxidizing agent and a secondary pollutant produced by nitrogen oxides and volatile organic compounds. Since humans are exposed to air pollutants primarily by inhalation, the respiratory system is one of the most important target organs of the harmful effects of air pollutants. In vitro studies have indicated exposure to variety of air pollutants may cause damage to lung, trachea, or bronchus, especially for fine particulate matter [1, 2]. Studies have shown that these pollutants cause lung inflammation, alveolar epithelial damage, and impaired mitochondrial function of the bronchial epithelial cells [2, 3]. They are potential candidates that induce COPD in the residents of Chiayi County.

Previous epidemiological studies on the relationship between exposure to traffic pollution sources and COPD have shown inconsistent conclusions. Andersen et al. [4] used a Cox proportional hazards model to study the association between COPD and exposure to traffic-related air pollution in Denmark. Their results showed that long-term exposure to traffic-related air pollution was associated with development of COPD. A cross-sectional study was conducted in Germany to investigate the effect of long-term exposure to PM_10_ from traffic on COPD. The results indicated that chronic exposure to PM_10_, NO_2_ and living near a major road might increase the risk of developing COPD and can have a detrimental effect on lung function [5]. Another cross-sectional study conducted in Southern Sweden also demonstrated that living close to traffic was associated with prevalence of COPD in adults [6]. However, a British nationwide cross-sectional study showed that close residential proximity to main roads did not increase the health risks of asthma, COPD, or allergic disease [7]. Likewise, another city-based British study showed that there was no evidence to suggest that living in close proximity to traffic is a major determinant of asthma, allergic disease, or COPD in adults [8]. Possible reasons for the different findings may be related to the assessment method of exposure concentration, culture differences as well as individual susceptibility to air pollution. Recently, studies have suggested that location-based air pollution data frequently have a far lower resolution than location-based health data. It is very likely to cause bias in assessing the relationship between exposure and response [9, 10].

One of the indicators for the development of COPD is decreasing lung function. A large-scale longitudinal cohort study conducted in Taiwan showed that for every 5 μg/m^3^ increase in PM_2.5_ concentration, forced vital capacity (FVC) decreased by 1.18%, and forced expiratory volume in 1 s (FEV_1_) decreased by 1.46%. Compared with the participants exposed to PM_2.5_ in the first quartile group, hazard ratio of developing COPD in the fourth quartile group was 1.23 (95% CI 1.09–1.39) [11]. Another population-based UK Biobank study found that exposure to PM_2.5_ is associated with decreased lung function and increased COPD prevalence, indicating that exposure to PM_2.5_ is a risk factor for COPD [12]. Furthermore, a population-based cohort study in the United States demonstrated that even at relatively low levels, long-term exposure to traffic and PM_2.5_ decrease FEV_1_ and FVC. In addition, it accelerated the rate of lung function decline [13]. Therefore, further study the relationship between exposure to traffic pollution and COPD is suggested.

Findings from recent studies indicate that outdoor air pollution and some health outcomes have a nonlinear exposure–response relationship [14–16]. Therefore, in this study, we used the hybrid kriging/land-use regression (LUR) model to obtain the levels of traffic-related pollutants (PM_2.5_ and O_3_) and further explored the effects of these pollutants on COPD risk in a large-scale community-based population under different study designs and statistical analysis. In addition, we adopted the distributed lag nonlinear model (DLNM) in the analysis of non-linear relationship and search for potential index for PM_2.5_ as well as O_3_ increments. Also, we evaluated the roles that traffic-related factors played in the association between air pollutants and COPD risk, such as road area as well as the traffic load of different kinds of vehicles. Considering that smoking is a significant risk factor for COPD and increased exposure to PM_2.5_; we executed a sensitivity analysis and approve the above results in the non-smokers.

## Materials and methods

### Study area and participants

**Fig. 1:**
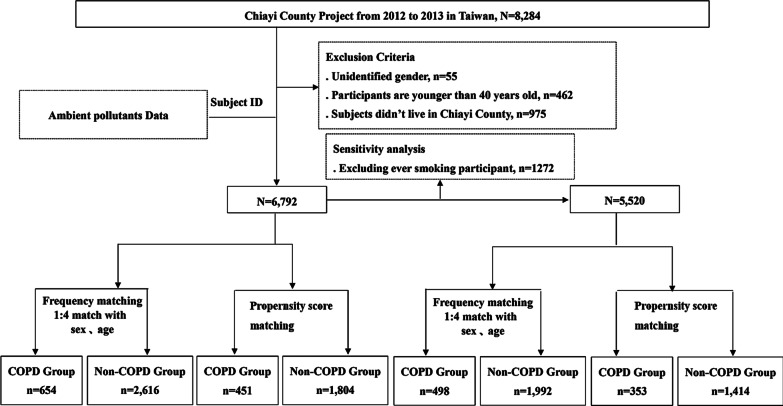
Study protocol of recruitment in this study

The study population was derived from a Chiayi County Complex Health Screening (CCHS) program launched from 2012 to 2013 in Chiayi County, which comprises 18 townships and has the highest percentage of elderly people (18.6%) in Taiwan. This program aimed to explore the effects of air pollution on long-term health risk for a community-based general population ≥ 40 years. Community recruitment was conducted during 2012 and 2013 and all residents aged ≥ 40 years lived in Chiayi County were invited to participate in this health program by mailing leaflet. A total of 8284 community residents voluntarily participated in the study. As shown in Fig. 1, we excluded participants younger than 40 years of age (N = 462), those who did not live in Chiayi County (N = 975), and records with missing sex data (N = 55). The final analytic cohort consisted of 6792 participants. According to the previous Epidemiology and Impact of COPD (EPIC) Asia survey, prevalence of COPD in Taiwan was approximately 10% [17]. About 3,416 of sample size are needed for all population aged ≥ 40 years old in Chiayi (N = 280,000) to have a confidence level of 95% that the real value is within ± 1% of the surveyed value. Therefore, our sample size is large enough to represent the population in Chiayi County. Written informed consent was obtained from all participants. This study was approved by the Research Ethics Committee of China Medical University Hospital, Taichung, Taiwan (DMR101-IRB061).

### Collection of questionnaires and health examinations

Well-trained personnel carried out standardized personal interviews based on a structured questionnaire, which contained demographic data and lifestyle variables contained cigarette smoking and quantity of areca nut chewing, consumption of alcohol and other beverages, participation in sport, consumption of three regular meals per day, and personal and familial history of cancer or other related diseases. In addition, all study population received health checkups, including baseline anthropometric and general biochemistry examinations such as blood pressure, plasma levels of triglycerides, total cholesterol, low-density lipoprotein cholesterol (LDL-C), high-density lipoprotein cholesterol (HDL-C), blood glucose, and blood creatinine after an 8-h fasting period. The biochemical data and self-reported data in the questionnaires were combined. Diabetes was defined as fasting glucose ≥ 126 mg/dL or use of insulin or oral hypoglycemic medications; hypertension was defined as systolic blood pressure (SBP) ≥ 140 mmHg, diastolic blood pressure (DBP) ≥ 90 mmHg, or antihypertensive medication use. Hyperlipidemia was defined as total cholesterol ≥ 200 mg/dL or triglyceride ≥ 130 mg/dL or confirmed disease status. In addition, baseline information about demographic characteristics and lifestyle variables was acquired from the questionnaires. Quantified (yes/ no) or frequency (numbers/ week) data of cigarette smoking and areca nut chewing, consumption of alcohol and other beverages, sport and three regular meal habits, personal and familial history of cancer or other related diseases were also collected. The prevalent cases of heart diseases, arthritis, asthma, chronic kidney diseases, and cancer were defined if they self-reported “Yes” to “Have you ever had heart diseases?” in the medical history portion of the questionnaire.

### Outcome Assessment and sensitivity analysis

COPD diagnoses were identified with ICD-9-CM codes (491, 492, and 496) through linkage of Taiwan National Health Insurance Research Databases (TNHIRD); almost all residents (> 99%) in Taiwan have been joined this TNHIRD program since 1995. The accuracy of COPD diagnosis recorded in the NHIRD has been validated [18], which indicating the accuracy of diagnoses was excellent. There were 668 COPD cases in the original population (N = 6792). We then adopted 1:4 frequency-matching with age and gender to the non-COPD group; there were 654 COPD cases and 2616 non-COPD controls. In addition, we constructed a propensity-score-matched population of 451 COPD cases and 1804 controls by matching age, gender, categorized BMI, ethnicity, level of education, marital status, and COPD-related comorbidity, including hypertension, diabetes, hyperlipidemia, heart diseases, arthritis, asthma, CKD, and cancer. Given that smoking habit is an important risk factor for COPD and increased exposure to PM_2.5_, we excluded current or ever smokers (N = 1272) and performed a sensitivity analysis through the similar methods of 1: 4 frequency-matching and propensity-score-matching (Fig. 1).

### ***A hybrid kriging/land-use regression (LUR) model for PM***_***2.5***_***, and O***_***3***_*** estimation***

We adopted the air pollutant data (PM_2.5_ and O_3_) collected from 71 Taiwan Environmental Protection Administration air quality monitoring stations between 2006 and 2013 for PM_2.5_ and 2000–2013 for O_3_ to calibrate our previously developed hybrid kriging/LUR model [10, 19]. The model takes into consideration the land use patterns, such as green areas, major traffic roads, temple incense burning, and industrial areas to improve the accuracy of predicting the exposure concentration of pollutants. Besides the above factors, other important variables such as temperature, relative humidity, wind-related factors, and meteorological as well as topography factors were also considered in the sequent stepwise model selection. Variance inflation factor < 3 was selected for the collinearity test in the model and also the statistical criterion of stepwise variable selection used in this study was 0.1. Furthermore, to improve the accuracy of PM_2.5_ and O_3_ variation predictions, the LUR model included the predicted concentration level from the kriging interpolation as a variable. Therefore, the hybrid approach further included the kriging-based concentration estimations as a predictor variable to improve the prediction performance of LUR. For data validity, the cross-validated R^2^ and RMSE were 0.87 and 5.02 μg/m^3^ for PM_2.5_ and 0.70 and 0.04 μg/m^3^ for O_3_, respectively. Finally, we calculated the overall average values of PM_2.5_ and O_3_ of all residents from the earliest start year to their corresponding year of recruitment (2012 or 2013).

### Statistical analysis

Baseline characteristics of the COPD and non-COPD groups were compared using chi-square test for categorical variables. Multiple logistic regression models were used to evaluate the prevalence ratios (PRs) and 95% confidence interval (CI) for the associations between PM_2.5_, and O_3_ (exposure variables) with respect to the PRs of COPD (outcome variable). Statistically significant variables of COPD shown in Tables 1 and 2 were considered as covariate variables adjusted for by including them in final multiple logistic regression models. Also, two-pollutant models were used to assess the associations of PM_2.5_ with COPD; the two-pollutant models included O_3_. Because the effect of O_3_ on COPD risk disappeared after adjustment for PM_2.5_, we only explored the role of PM_2.5_ in the following analysis. Either b-spline or natural cubic was used for fitting the non-linear dose–response relationship of PM_2.5_ and COPD under different degrees of freedom. Selection of the degrees of freedoms in the final model was determined using the minimum Akaike information criterion (DLNM package in the R program). To determine the important factors for PM_2.5_ increments, we contained all LUR-related indices with different circular buffers (500 m, 1000 m, 1500 m, 2000 m, 2500 m, 3000 m, 4000 m, and 5000 m) in the model selection. These variables included normalized difference vegetation index (NDVI), area of industrial land, number of temples, distance from residence to municipal waste incinerator, area of traffic road. Among these variables, the overall value of R^2^ of the four indices including within a 5000 m circular buffer of NDVI, area of local road area, area of industrial land within a 4000 m circular buffer, and amounts of temples within a 500 m circular buffer, was approximately 0.75 for PM_2.5_. We further evaluated the interactions of LUR-related indices and levels of PM_2.5_ on PRs of COPD in the propensity-score-matched population using multiple logistic regression models. The cut-off values were defined as median of LUR-related indices and PM_2.5_ in the non-COPD groups. Furthermore, we analyzed the associations between the daily traffic load of different types of vehicles and the annual PM_2.5_ level at traffic load monitoring stations in Chiayi County during the study period by repeated measurements analysis. Different correlation structures of repeated data were all used in the generalized estimating equation approach with calculating Pan’s quasilikelihood under the independence model information criterion (QIC). The correlation structure with the lowest QIC score was generally considered to be the best [20]. All data were analyzed using the SAS statistical package (SAS, version 9.4, Cary, NC) and R software version 3.6.3. A two-sided p-value < 0.05 was considered statistically significant.

**Table 1:** Descriptive characteristics between study participants with COPD and without COPD

Variables	Frequency matching			Propensity-score matching		
	Case	Control	p-values	Case	Control	p-values
	n = 654	n = 2616		n = 451	n = 1804	
Age	66.00 ± 12.15	65.59 ± 12.16				
40–50	82 (12.54)	328 (12.54)	1.0000	63 (13.97)	237 (13.14)	0.6758
50–60	112 (17.13)	448 (17.13)		86 (19.07)	389 (21.56)	
60–70	172 (26.30)	688 (26.30)		127 (28.16)	460 (25.50)	
70–80	210 (32.11)	840 (32.11)		135 (29.93)	555 (30.76)	
≥ 80	78 (11.93)	312 (11.93)		40 (8.87)	163 (9.04)	
Sex						
Male	301 (46.02)	1204 (46.02)	1.0000	205 (45.45)	811 (44.96)	0.8489
Female	353 (53.98)	1412 (53.98)		246 (54.55)	993 (55.04)	
BMI (Unit = 3.69)						
Underweight	22 (3.38)	52 (2.00)	0.0840	9 (2.00)	40 (2.22)	0.8965
Ordinary	262 (40.31)	1078 (41.49)		191 (42.35)	794 (44.01)	
Overweight	188 (28.92)	816 (31.41)		142 (31.49)	541 (29.99)	
Obesity	178 (27.38)	652 (25.10)		109 (24.17)	429 (23.78)	
Ethnicity						
Holo Taiwanese	546 (96.47)	2397 (96.73)	0.9405	434 (96.23)	1754 (97.23)	0.5352
Hakka Taiwanese	9 (1.59)	35 (1.41)		7 (1.55)	21 (1.16)	
Mainland Chinese	11 (1.94)	46 (1.86)		10 (2.22)	29 (1.61)	
Education						
Elementary school or below	401 (63.05)	1558 (60.67)	0.5312	272 (60.31)	1099 (60.92)	0.5820
High school	170 (26.73)	738 (28.74)		119 (26.39)	440 (24.39)	
College or above	65 (10.22)	272 (10.59)		60 (13.30)	265 (14.69)	
Marriage						
Married	534 (83.31)	2209 (86.83)	0.0191	384 (85.14)	1577 (87.42)	0.3291
Single	20 (3.12)	43 (1.69)		12 (2.66)	50 (2.77)	
Widowed /divorce	87 (13.57)	292 (11.48)		55 (12.20)	177 (9.81)	
Hypertension	414 (63.79)	1582 (61.08)	0.2044	261 (57.87)	1068 (59.20)	0.6075
Diabetes	75 (11.68)	347 (13.50)	0.0076	84 (18.63)	302 (16.74)	0.3419
Hyperlipidemia	392 (61.15)	1669 (65.17)	0.0576	279 (61.86)	1135 (62.92)	0.6791
Heart disease	94 (14.62)	268 (10.46)	0.0029	50 (11.09)	179 (9.92)	0.4642
Arthritis	107 (16.77)	306 (11.95)	0.0012	57 (12.64)	173 (9.59)	0.0557
Asthma	67 (10.47)	63 (2.46)	< 0.0001	13 (2.88)	54 (2.99)	0.9013
CKD	18 3 (28.33)	728 (28.18)	0.9421	108 (23.95)	418 (23.17)	0.7274
Cancer	18 (2.81)	37 (1.44)	0.0170	9 (2.00)	33 (1.83)	0.8153

**Table 2:** Distributions of lifestyles- and dietary-related factors between study participants with COPD and without COPD

Variables	Frequency matching			Propensity-score matching		
	Case	Control	p-values	Case	Control	p-values
	n = 654	n = 2,616		n = 451	n = 1,804	
Smoking						
Never	498 (76.62)	2120 (81.76)	0.0030	340 (75.56)	1478 (82.11)	0.0016
Ever	152 (23.38)	473 (18.24)		110 (24.44)	322 (17.89)	
Alcohol drinking						
No	525 (80.89)	2192 (84.54)	0.0243	370 (82.59)	1539 (85.60)	0.1109
Yes	124 (19.11)	401 (15.46)		78 (17.41)	259 (14.40)	
Tea drinking						
No	465 (71.76)	1824 (70.51)	0.5307	302 (67.41)	1232 (68.67)	0.6070
Yes	183 (28.24)	763 (29.49)		146 (32.59)	562 (31.33)	
Coffee drinking						
No	604 (93.21)	2382 (92.22)	0.3939	418 (93.30)	1639 (91.36)	0.1811
Yes	44 (6.79)	201 (7.78)		30 (6.70)	155 (8.64)	
Betel consumption						
No	575 (89.01)	2396 (92.51)	0.0037	397 (88.62)	1660 (92.27)	0.0128
Yes	71 (10.99)	194 (7.49)		51 (11.38)	139 (7.73)	
Sugary drink (bottle/week)						
< 3	574 (92.13)	2219 (90.53)	0.2191	405 (93.75)	1593 (92.40)	0.4827
3–7	37 (5.94)	153 (6.24)		20 (4.63)	87 (5.05)	
≥ 7	12 (1.93)	79 (3.22)		7 (1.62)	44 (2.55)	
Vegetables consumption (bowl /day)						
< 1	258 (39.81)	970 (37.39)	0.3079	180 (40.00)	648 (36.06)	0.2776
1–3	331 (51.08)	1411 (54.39)		231 (51.33)	994 (55.31)	
≥ 3	59 (9.10)	213 (8.21)		39 (8.67)	155 (8.63)	
Fruit consumption (bowl /day)						
< 1	391 (60.25)	1506 (58.06)	0.5457	269 (59.65)	1023 (56.93)	0.5533
1–3	218 (33.59)	931 (35.89)		154 (34.15)	662 (36.84)	
≥ 3	40 (6.16)	157 (6.05)		28 (6.21)	112 (6.23)	

## Results

### Basic characteristics and lifestyle-related variables in COPD and non COPD population

Descriptive characteristics between study participants with and without COPD are shown in Table 1. There was approximately a 1:1 sex ratio of males to females, with average age of 66 years. About half of the entire study population had a BMI that was categorized as overweight or obesity, 62% had an elementary level education or below, and 85% were married.

For comorbidities, the study population with COPD had a higher prevalence of diabetes, heart diseases, arthritis, asthma, and cancer (all p-values < 0.05). In addition, we constructed a propensity-score analysis, matching the study population with similar distributions of COPD-related variables described above between the COPD and non-COPD groups.

The associations between lifestyles- and dietary-related variables and prevalent rate ratios of COPD are shown in Table 2. Approximately 10%-30% of the study population had habits of cigarette smoking, alcohol consumption, tea and coffee drinking, and betel consumption. Most of the subjects reported consuming less than 3 bottles of sugary drinks per week. Half of study population consumed one to three bowls of vegetables per day; however, about 60% had less than one bowl of fruit per day. Cigarette smoking and betel consumption were significantly associated with COPD irrespective of age and sex matching as well as propensity-score matching. In addition, through age and gender matching, there was a significant difference in the distribution of alcohol drinking between the COPD group and the control group. However, by way of propensity score matching, this significant difference disappeared.

### Linear and non-linear relationships of PM_2.5_, O_3_, and prevalence risk of COPD

**Fig. 2:**
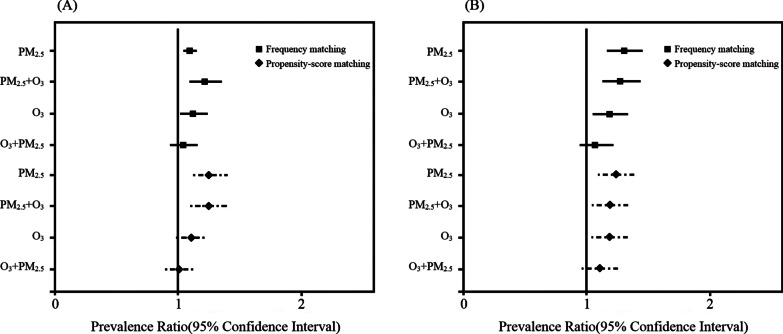
Associations between exposure to air pollution (PM_2.5_ and O_3_) and prevalence ratios of COPD in population with frequency matching (Black square) and with propensity-score matching (Black diamond) analyzed by single-pollutant model and two-pollutant model. **A** all population (**B**) non-smokers in the sensitivity analysis

We further explored the associations between exposure to air pollution and prevalence ratios of COPD by adjusting for risk factors shown in Tables 1 and 2. The results are presented in Fig. 2. The increased prevalence ratios of COPD per SD increment of PM_2.5_ were 1.10 (95% CI 1.05–1.15), and 1.25-fold (95% CI 1.13–1.40) in the population with age and sex matching as well as propensity-score matching, respectively (Fig. 2A). The significant results were also found in two-pollutant models, which included the levels of O_3_. In addition, for O_3_, there was about 11–12% significantly increased prevalence ratios of COPD per SD increment of O_3_ in two kinds of matching population; however, after adjusting for PM_2.5_ in two-pollutant models, the effects of O_3_ on COPD were not found. The detailed data of the above prevalence ratios are shown in Additional file 1: Table S1.

In the sensitivity analysis, we excluded the ever smokers from the original cohort of 6792 residents and performed a similar 1:4 matching to repeatedly explore the association between exposure to air pollution (PM_2.5_ and O_3_) and COPD. The results indicated that there were significant positive associations between levels of PM_2.5_ and COPD, irrespective of whether a single or two-pollutant mode was used (Fig. 2B). For two-pollutant models, there were significantly 1.25-fold and 1.19-fold risks of COPD prevalence (both p < 0.05).

**Fig. 3:**
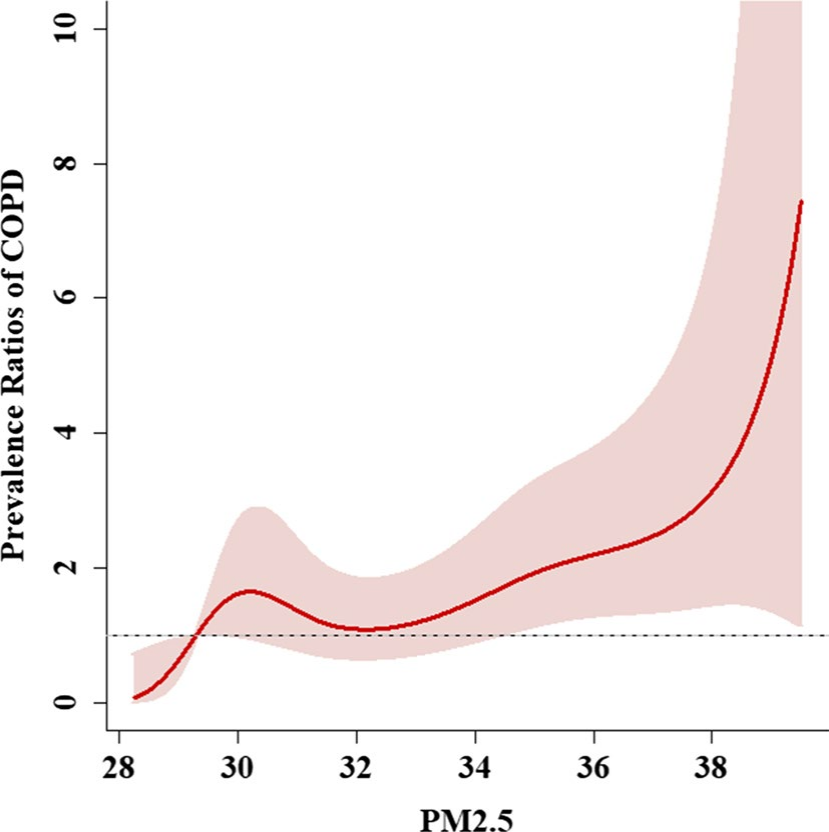
Non-linear relationships of PM_2.5_ levels and COPD in the propensity-score matching population

We further executed the DLNM analysis, which was set as the simple b-splines function with five degrees of freedom and with the minimum AIC value (2198.85) (Additional file 1: Table S2). Also, we selected the lowest 5% of PM_2.5_ levels as a reference value and the results showed a non-linear dose–response relationship of significant COPD prevalence and PM_2.5_ above 35 μg/m^3^ among propensity-score matching population. The spline results are displayed graphically in Fig. 3.

### The role of traffic-related variables for PM_2.5_ and prevalence ratios of COPD

Since the novelty effect of PM_2.5_ was observed in the above results compared to O_3_, we only explored the role of PM_2.5_ in the following analysis. For the four important contributors of PM_2.5_ increment from model selection (NDVI, area of industrial land, number of temples, and area of traffic road) we evaluated the interactions of individual contributor and PM_2.5_ levels on prevalence ratios of COPD in the propensity score matched population in Table 3. The results suggest statistically significant dose–response relationships between increasing levels of PM_2.5_ combined with increased amount of individual contributor for positive associations of COPD (all p < 0.01). Among the four aforementioned contributors, high PM_2.5_ levels combined with high road area or with high area of industrial land significantly interact on increased prevalence ratios of COPD (both p < 0.01) after adjusting for smoking and betel consumption. Furthermore, we calculated the nearest distance from residential address to the surrounding bus station and analyzed the associations between the levels of PM_2.5_ and distance to bus station through generalized linear regression model. For residents that lived within 1 km of the bus station, the results suggested residents who live farther away from the bus station have a low PM2.5 concentration after adjustment for townships and smoking habit (p = 0.004, data not shown).

**Table 3:** Interactions of air pollutants, and LUR-related variables on the PRs of COPD in propensity-scoring matched population

LUR-related variables	PM_2.5_ (μg/m^3^)	OR (95% CI)^a^	*p *_Interaction_
NDVI			0.28
< 0.45	< 35	Reference	
≥ 0.45	< 35	0.99 (0.75–1.30)	
< 0.45	≥ 35	1.32 (0.81–2.15)	
≥ 0.45	≥ 35	1.79 (1.39–2.29) ^**^	
		*p*_Trend_ < 0.0001	
Area of industrial land (m^2^/ grid)			< 0.01
< 18.2	< 35	Reference	
≥ 18.2	< 35	0.90 (0.69–1.18)	
< 18.2	≥ 35	1.18 (0.85–1.63)	
≥ 18.2	≥ 35	2.18 (1.61–2.94) ^**^	
		*p*_Trend_ < 0.0002	
Road area (m^2^)			< 0.01
< 20.5	< 35	Reference	
≥ 20.5	< 35	0.78 (0.60–1.02)	
< 20.5	≥ 35	1.09 (0.79–1.50)	
≥ 20.5	≥ 35	2.05 (1.53–2.76) ^**^	
		*p*_Trend_ < 0.0001	
Number of temples (*10^6^ per m^2^)			0.34
< 0	< 35	Reference	
≥ 1	< 35	0.92 (0.67–1.27)	
< 0	≥ 35	1.96 (1.48–2.60) ^**^	
≥ 1	≥ 35	1.44 (1.08–1.93) ^*^	
		*p*_Trend_ = 0.0001	

SD, standard deviation; NDVI, Normalized Difference Vegetation Index. ^a^Multiple logistic regressions included confounding factors of cigarette smoking and betel consumption

^*^p < 0.05, ^**^p < 0.01

For the role of area of traffic road on PM_2.5_ increment, we further analyzed the associations between the daily traffic load of different types of vehicles and annual PM_2.5_ at traffic flow monitoring stations in Chiayi County during 2009–2014 using repeated measurements analysis. The results showed an unstructured correlation structure had the lowest QIC score. It indicated a statistically significant positive association between traffic load and increased levels of PM_2.5_, especially for passenger cars, buses, and trucks (p < 0.01, p < 0.01, and p < 0.01, respectively) (Table 4). It demonstrated that the PM_2.5_ concentrations in Chiayi County were significantly related to emissions from sedans, buses, and trucks.

**Table 4:** Association between PM_2.5_ levels and daily traffic load of different type of vehicles at traffic station in Chiayi County through the generalized estimating equation approach

Car type	Mean (SD)	Min–Max
Motorcycle (10,000/day)	0.25 (0.32)	0–1.53
Sedan (10,000/day)	0.92 (0.71)	0.05–2.84
Bus (1000/day)	0.16 (0.13)	0–0.7
Truck (1000/day)	0.54 (0.52)	0.01–1.93

	Unstructured structure				Exchangeable structure				Autoregressive structure			
Car type	β	SE	p-value	QIC	β	SE	p-value	QIC	β	SE	p-value	QIC
Motorcycle	− 0.66	0.55	0.23	1880.28	0.65	0.61	0.29	1930.55	1.40	0.58	0.02	1920.68
Sedan	1.00	0.19	< 0.01	190.85	− 0.10	0.33	0.76	194.36	0.26	0.34	0.44	194.13
Bus	5.48	1.75	< 0.01	190.92	− 0.64	1.44	0.66	193.61	1.97	1.25	0.16	192.38
Truck	0.98	0.18	< 0.01	190.50	− 0.46	0.54	0.39	195.78	− 0.57	0.43	0.18	193.60

## Discussion

This present study adopted the hybrid kriging/LUR model included the kriging-based concentration estimations as a predictor variable to improve the prediction performance of LUR and provided the individual level of exposure to ambient air pollutants. The results indicated a positive correlation between exposure to PM_2.5_ and PRs of COPD in a community-based population even excluding all smokers from the original cohort. Besides, traffic-related variables including road area, traffic load of sedans, buses, and trucks, as well as living near a bus station were relative to PM_2.5_ increment.

Several epidemiological studies have shown that exposure to PM_2.5_ is related to COPD. It is estimated that long-term exposure to ambient PM_2.5_ contributes between 10.7% and 15.3% to COPD in Iran [21]. A meta-analysis study demonstrated that exposure to PM_2.5_ is significantly associated with prevalence of COPD (OR: 2.32, 95% CI 1.91–2.82) [22]. A Korean study showed that concentration of PM_2.5_ was associated with increased COPD-related hospital visits in Chuncheon [23]. The results of this investigation are consistent with those of the above studies. In addition, we used the DLNM model to analyze the dose–response relationship between exposure to PM_2.5_ and prevalence of COPD. Our results demonstrated that there was a non-linear relationship between PM_2.5_ and prevalence of COPD. As the concentration of PM_2.5_ rose above 35 μg/m^3^, the prevalence of COPD increased significantly. This finding is very similar to another Taiwanese population-based study, which found that exposure to PM_2.5_ at concentrations greater than 38.98 μg/m^3^ increased susceptibility to COPD among nonsmokers [24].

The present study applied the hybrid kriging/LUR model to predict the exposure concentration of air pollutants at residential addresses. This model has been used extensively to assess the effects of exposure to PM_2.5_ on human health [25–27]. In the model, we consider the NDVI, the areas of the industrial land, the areas of the traffic road, and the number of temples, etc. to predict the exposure concentration of PM_2.5_ and ozone. We found that increased levels of PM_2.5_ were significantly associated with NDVI, areas of industrial land, traffic roads, and number of temples. Among them, the areas of industrial parks and traffic roads near the subject’s residency and the exposure concentration of PM_2.5_ have a multiplicative effect on the PRs of COPD. The results demonstrated that compared with the low traffic road area and low PM_2.5_ concentration, the population with high traffic road area and a high PM_2.5_ concentration near their homes had a 1.66 times greater risk (95% CI 1.21–2.28) of developing COPD. In addition, we verified this association by calculating the nearest distance from the residential address of subjects to surrounding bus stations. A statistically significant negative association between the distance and levels of PM_2.5_ was observed (data not shown). The number of vehicles in Chiayi County increasing yearly is considered to be correlated to gradually increasing the risk of respiratory diseases in Chiayi County; this is even higher than the situation in Taiwan (Taiwan Ministry of Health and Welfare, 2020). Our data show that traffic is one of the main factors contributing to the increasing development of COPD in Chiayi County.

We used the data from the traffic flow monitoring stations (N = 74) in Chiayi County to analyze the relationship between the daily traffic flow of various vehicles and the concentration of PM_2.5_ to explore the impact of different types of vehicles on the concentrations of PM_2.5_. Our results show that buses had the greatest impact on the variability of PM_2.5_ concentration, followed by sedans, and then trucks. The relationship between the traffic load of these three types of vehicles and PM_2.5_ showed a statistically significant correlation. Studies have suggested that diesel-fueled vehicles have a higher PM_2.5_ emission factor than that of gasoline-fueled vehicles. For example, a study in Taiwan showed that a sedan's emission factor is 1.25 mg/km, while those of diesel engine trucks are as high as 185 mg/km [28]. Another Chinese study also showed that the emission factor for diesel-fueled vehicles is about 257 mg/km and for non-diesel-fueled vehicles is about 17 mg/km [29]. Both buses and trucks are diesel-fueled vehicles. Therefore, the findings of our study showed that the traffic flow of the buses and trucks had a positive correlation with the concentration of PM_2.5_. Since buses need to allow passengers to get on and off within a certain distance, the operations of their engines vary greatly in such conditions, which may be attributed to buses having the greatest impact on PM_2.5_ emissions.

Previous large-scale community-based studies suggest there are high frequencies of additional comorbidities among elderly people with COPD [30, 31], which is consistent with our findings. A survey from the US Center for Disease Control and Prevention assistance found that the most common co-morbid chronic conditions among people with COPD included arthritis, asthma, cancer, and coronary artery disease [32]. Oxidative stress and systemic inflammation were the common mechanisms in the development and progression of COPD as well as other comorbidities [33, 34]. COPD is a state of systemic inflammation [35] and high levels of inflammatory markers are associated with the severity of airflow obstruction and cardiac injury [36]. Hypoxia induced expression of proinflammatory transcription factors lead to endothelial dysfunction and then to atherosclerosis [37]. Lung infections were very important precipitating factors for acute exacerbation of COPD, and some of them accelerated atherosclerosis and precipitated acute coronary syndrome by causing plaque instability [38]. COPD also had a detrimental effect on CAD through hypoxia, decreased respiratory muscle strength, and use of bronchodilators [39]. Inflammatory mediators, such as IL-17 and anti–citrullinated protein antibodies, which play a role in arthritis, are also involved in the pathogenesis of COPD [40]. COPD, arthritis, and coronary artery disease share many of the same risk factors, such as sex, age, tobacco use, obesity, and sedentary lifestyle [41]. The abovementioned mechanisms must be considered to explain the association between COPD, CAD, and arthritis.

Our study has several limitations. We could not exclude the temporality of the association between air pollution and COPD. This may overlook pre-existing lung injury attributed to occupational exposure or lifestyle factors such as smoking and increased sensitivity to exposure to air pollution. In addition, empirical duration in outdoor or indoor was not acquired in the analysis. However, smoking is an important source of indoor exposure to air pollution. Therefore, we constructed a sensitivity analysis to excluded the possible effect of smoking and still found a positive association between levels of PM_2.5_ and prevalence ratios of COPD. Another limitation is that the study population was volunteers participating in a community-based health screening; therefore, most participants tended to be healthy and this could underestimate the prevalence ratios of COPD. In addition, study participants without sever COPD did not seek medical help and could not be acquired in our study, which may bias the present results. Moreover, about 70% of study population was elder with average aged 65 years old (in Table 1) and they had no work. The data of outdoor activities duration were not well evaluated for elder population. Therefore, these variables were not further considered in our study. We did not collect chemical composition data of PM_2.5_ such as polycyclic aromatic hydrocarbons or heavy metals in this study. However, it is suggested that PAHs or heavy metals in PM_2.5_ may have a positive association with development of COPD [42, 43]. We recommended that follow-up studies could further analyze the impact of PM_2.5_ components on COPD to understand the mechanisms of PM_2.5_ on COPD.

## Conclusion

The positive association between levels of PM_2.5_ and PRs of COPD was observed in a community-based population even excluding all smokers from the original cohort. The results showed a non-linear relationship of PM_2.5_ above 35 μg/m^3^ and prevalence of COPD in the DLNM analysis. Areas of industrial land as well as roads individually interacted with levels of PM_2.5_ on increased prevalence ratios of COPD. In the analysis of traffic flow, we found that sedans, buses, and trucks all had a significant positive association with the variations in PM_2.5_ concentration. It is suggested that traffic emitted PM_2.5_ is an important factor in the development of COPD in Chiayi County. In the future, empirical measurements of indoor and outdoor air pollution should be further expanded and explored with regard to the role of air pollution on COPD incidence.

## Supplementary Information

**Additional file 1: Table S1. **Associations between indices of air pollutants and COPD risk: single- and two-pollutant models. **Table S2**. AIC values under different spline functions and degree of freedom.

## Abbreviations

COPD: Chronic obstructive pulmonary disease
PM_2.5_: Particulate matter_2.5_
NO_2_: Nitrogen dioxide_2_
O_3_: Ozone
PRs: Prevalence ratios
LDL-C: Low-density lipoprotein cholesterol
HDL-C: High-density lipoprotein cholesterol
SBP: Systolic blood pressure
DBP: Diastolic blood pressure
TNHIRD: Taiwan National Health Insurance Research Databases
LUR: Hybrid kriging/land-use regression
NDVI: Normalized difference vegetation index
