# Supplementary material for: The effects of traffic-related air pollutants on chronic obstructive pulmonary disease in the community-based general population
Source: Respir Res. 2021 Aug 3;22:217. doi: 10.1186/s12931-021-01812-x (PMC8336021; doi:10.1186/s12931-021-01812-x)
Supplement: Supplementary file 1 — Additional file 1: Table S1. Associations between indices of air pollutants and COPD risk: single- and two-pollutant models. Table S2. AIC values under different spline functions and degree of freedom. [file 12931_2021_1812_MOESM1_ESM.docx]

| Table S1 Associations between indices of air pollutants and COPD risk: single- and two-pollutant models | | | | | | |
| --- | --- | --- | --- | --- | --- | --- |
|  | All population | | | Sensitivity analysis (Non-smokers) | | |
|  | Frequency matching | Propensity-score matching |  | Frequency matching | Propensity-score matching |  |
| Air pollutants | PR (95%CI) ^a^ | PR (95%CI) ^b^ |  | PR (95%CI) ^c^ | PR (95%CI) ^d^ |  |
| PM_2.5_ (per SD increment) (μg/m^3^) | 1.10 (1.05-1.15) ^**^ | 1.25 (1.13-1.40) ^**^ |  | 1.28 (1.15-1.43) ^**^ | 1.24 (1.10-1.40) ^**^ |  |
| + O3 | 1.22 (1.10-1.36) ^**^ | 1.25 (1.11-1.40) ^**^ |  | 1.25 (1.11-1.41) ^**^ | 1.19 (1.04-1.35) ^*^ |  |
| O3 (per SD increment) (ppb) | 1.12 (1.02-1.24) ^*^ | 1.11 (0.99-1.23) ^#^ |  | 1.16 (1.03-1.30) ^*^ | 1.20 (1.06-1.36) ^**^ |  |
| + PM_2.5_ | 1.04 (0.93-1.16) | 1.01 (0.90-1.14) |  | 1.05 (0.93-1.19) | 1.12 (0.94-1.28) |  |

SD: standard deviation; ^a^ Multiple logistic regressions included confounding factors of marriage, diabetes, heart disease, arthritis, asthma, cancer, smoke, alcohol drinking, and betel consumption; ^b^ Multiple logistic regressions included confounding factors of smoke, and betel consumption. ^c^ Multiple logistic regressions included confounding factors of BMI, asthma, coffee deinking, and heart Disease. ^d^ Multiple logistic regressions included confounding factors of betel consumption. ^**^ P<0.01; ^*^ 0.01<P<0.05; ^#^ 0.05<P<0.1

| Table S2 AIC values under different spline functions and degree of freedom | | |
| --- | --- | --- |
| Spline function | degree of freedom | AIC |
| Natural spline | 3 | 2199.666 |
|  | 4 | 2200.243 |
|  | 5 | 2200.669 |
|  | 6 | 2201.547 |
|  | 7 | 2201.34 |
|  | 8 | 2201.179 |
|  | 9 | 2202.436 |
|  |  |  |
| Basis spline | 3 | 2199.421 |
|  | 4 | 2200.986 |
|  | 5 | 2198.85 |
|  | 6 | 2199.963 |
|  | 7 | 2201.467 |
|  | 8 | 2202.255 |
|  | 9 | 2203.441 |
